# When Cellulitis Is Not Cellulitis: A Case of Suspected Mycosis Fungoides With Nondiagnostic Biopsies

**DOI:** 10.7759/cureus.107086

**Published:** 2026-04-15

**Authors:** Alireza Izadian Bidgoli, Jordan De Guzman, Carolina Karuppiah, Samantha Abbott, Alberto Gomez Veliz

**Affiliations:** 1 Internal Medicine, American University of the Caribbean School of Medicine, Cupecoy, SXM; 2 Internal Medicine, Jackson Memorial Hospital, Miami, USA

**Keywords:** cellulitis mimicker, clinicopathologic discordance, cutaneous t-cell lymphoma, diagnostic delay, mycosis fungoides

## Abstract

Mycosis fungoides, the most common subtype of cutaneous T-cell lymphoma, is characterized by an indolent course and a wide spectrum of clinical presentations that frequently mimic benign inflammatory dermatoses. Early-stage disease is particularly challenging to diagnose, often requiring repeated clinicopathologic correlation due to nonspecific histopathologic findings. We present the case of a 64-year-old female with a several-year history of diffuse pruritic skin lesions who was admitted for acute right lower extremity pain, erythema, and swelling concerning cellulitis. While her acute symptoms improved with antibiotic therapy, dermatologic evaluation revealed chronic, widespread hypopigmented and scaly patches and plaques, raising concern for underlying cutaneous T-cell lymphoma. Multiple prior and current skin biopsies demonstrated subacute spongiotic dermatitis without definitive evidence of lymphoma, and immunophenotypic studies, including flow cytometry, were nondiagnostic. This case highlights the diagnostic challenge of suspected early-stage mycosis fungoides, particularly when histopathologic findings are inconclusive and clinical features overlap with common conditions such as cellulitis. The coexistence of an acute inflammatory process may further obscure recognition of an underlying cutaneous lymphoproliferative disorder. Recognition of persistent, atypical, or treatment-refractory dermatologic findings should prompt continued evaluation and consideration of repeat biopsy. This case underscores the importance of maintaining clinical suspicion and integrating longitudinal clinical and pathologic data in the diagnosis of cutaneous T-cell lymphoma.

## Introduction

Cellulitis is a frequently encountered clinical diagnosis characterized by the acute onset of localized erythema, warmth, swelling, and tenderness, and is commonly managed empirically in both inpatient and outpatient settings [1,2]. Despite its prevalence, clinical diagnosis remains inherently imprecise, with misdiagnosis rates reported to approach 30%, as a substantial proportion of cases initially labeled as cellulitis ultimately represent noninfectious mimickers, including inflammatory dermatoses, vascular conditions, and malignancies [1,2]. This diagnostic ambiguity creates a risk of anchoring bias and may delay recognition of alternative underlying pathology [1,2].

Mycosis fungoides (MF), the most common subtype of cutaneous T-cell lymphoma, is an indolent malignancy of skin-homing CD4+ T lymphocytes (a type of immune cell involved in adaptive immunity) that exhibits a broad spectrum of clinical presentations [3-5]. Early-stage disease classically manifests as patches and plaques, flat or raised areas of skin involvement, but is well recognized for its ability to mimic benign inflammatory dermatoses such as eczema, psoriasis, and contact dermatitis, frequently resulting in prolonged diagnostic delay [3-5]. The median time from symptom onset to diagnosis has been reported to span several years, reflecting both clinical and histopathologic ambiguity [3,5-7].

Histopathologic evaluation in early MF is often nondiagnostic, with findings that may overlap with common inflammatory conditions such as spongiotic dermatitis (a pattern of skin inflammation characterized by intercellular edema in the epidermis) [3,4,8]. As a result, definitive diagnosis frequently requires repeated biopsies and integration of clinical, histologic, and immunophenotypic data over time [3,4,8]. Even with advanced diagnostic techniques, early disease may lack definitive abnormalities, further complicating diagnostic certainty [3,4,8].

The diagnostic challenge is further amplified when chronic cutaneous disease coexists with an acute inflammatory process [9-11]. Superimposed conditions such as cellulitis may reinforce an initial diagnosis of infection, obscuring recognition of an underlying dermatologic or lymphoproliferative disorder [9-11]. Case reports have demonstrated that MF may initially be misdiagnosed as cellulitis or other inflammatory conditions, resulting in delayed or inappropriate management [9-11].

## Case presentation

Clinical presentation

A 64-year-old female with a past medical history significant for chronic deep vein thrombosis on anticoagulation, hypertension, dyslipidemia, and heart failure with preserved ejection fraction presented with progressive right lower extremity pain, swelling, and functional decline. The patient reported worsening discomfort over several days, accompanied by difficulty ambulating and increasing lower extremity edema. She denied recent trauma but endorsed progressive fatigue and generalized weakness. Notably, the patient had limited prior engagement with the healthcare system due to financial constraints and lack of consistent access to care, which contributed to delayed evaluation of her symptoms.

On presentation, she was hemodynamically stable but appeared chronically ill. Physical examination demonstrated diffuse swelling of the right lower extremity with associated tenderness and overlying skin changes concerning cellulitis. Cutaneous findings included areas of induration and abnormal skin texture, raising concern for an underlying dermatologic or infiltrative process beyond simple infection.

Evaluation (history, examination, laboratory findings, and imaging)

Laboratory evaluation demonstrated preserved renal and metabolic function, with stable electrolyte levels throughout hospitalization. Inflammatory markers were elevated, with C-reactive protein rising from 1.4 to 3.7 mg/dL, consistent with a superimposed inflammatory or infectious process. Mild hypoalbuminemia (3.2-3.3 g/dL) was noted, which may reflect chronic disease and systemic inflammation. Liver function tests showed transient mild elevations in transaminases, which normalized during the hospital course. Overall, laboratory findings did not demonstrate clear evidence of systemic organ involvement or high tumor burden; however, these findings are limited in their ability to exclude early or occult systemic disease, particularly in the absence of a definitive diagnosis. A detailed summary of laboratory findings is provided in Table 1.

**Table 1:** Summary of laboratory findings at presentation and during hospitalization.

Laboratory test	At presentation	During hospitalization	Reference range (units)
Glucose	84	127	70–100 mg/dL
Sodium	139	136–140	135–145 mmol/L
Potassium	4.4	3.9–4.0	3.5–5.0 mmol/L
Chloride	106	101–107	98–107 mmol/L
Bicarbonate (CO₂)	26	26–28	22–29 mmol/L
Blood urea nitrogen (BUN)	14	9–14	7–20 mg/dL
Creatinine	0.54	0.50–0.60	0.6–1.3 mg/dL
Estimated glomerular filtration rate (eGFR)	>90	>90	>60 mL/minute
Calcium	9.6	8.7–9.1	8.5–10.5 mg/dL
Total protein	6.5	5.7–5.9	6.0–8.3 g/dL
Albumin	3.7	3.2–3.3	3.5–5.0 g/dL
Total bilirubin	0.3	0.3	0.1–1.2 mg/dL
Aspartate aminotransferase (AST)	23	46	10–40 U/L
Alanine aminotransferase (ALT)	19	33	7–56 U/L
Alkaline phosphatase	84	81–93	44–147 U/L
Magnesium	1.8	—	1.7–2.2 mg/dL
Phosphorus	4.7	—	2.5–4.5 mg/dL
C-reactive protein (CRP)	1.4	3.7	<0.5 mg/dL
Lactate dehydrogenase (LDH)	190	—	140–280 U/L

Given the patient’s persistent symptoms and concerning cutaneous findings, further systemic evaluation was pursued to assess for underlying malignancy or extracutaneous involvement. Cross-sectional imaging with contrast-enhanced CT of the chest, abdomen, and pelvis was obtained.

Contrast-enhanced CT imaging of the abdomen and pelvis did not reveal any acute intra-abdominal pathology or findings clinically significant to the patient’s presentation. Incidental findings, including diverticulosis without diverticulitis, a nonobstructing right renal calculus, a small renal cyst, and mild common bile duct dilation likely related to prior cholecystectomy, were noted. No significant lymphadenopathy or evidence of systemic involvement was identified.

**Figure 1:**
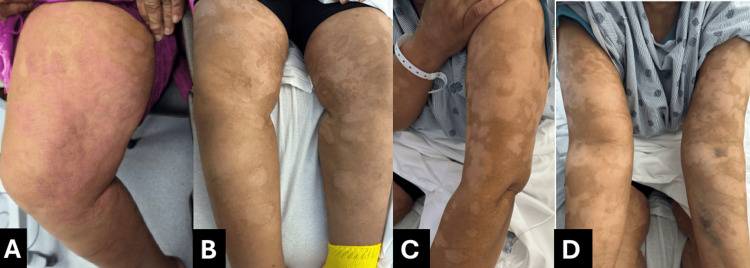
Cutaneous findings suspicious for mycosis fungoides presenting as diffuse pigmentary alteration. (A) Initial presentation demonstrating hypopigmented and hyperpigmented patches with areas of induration on the lower extremity. (B–D) Persistent patch and plaque morphology at discharge, highlighting chronicity and lack of complete resolution despite treatment of superimposed cellulitis.

**Figure 2:**
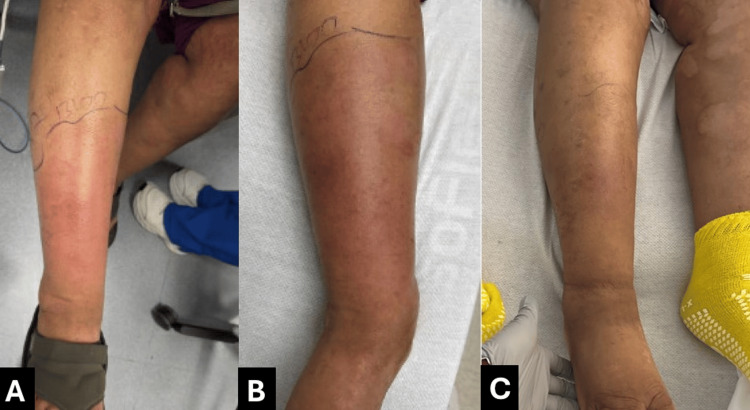
Diffuse erythema and swelling before and after treatment, consistent with superimposed cellulitis. (A) At admission: diffuse erythema, warmth, and swelling with poorly demarcated borders. (B) During treatment: partial improvement in erythema and edema. (C) At discharge: resolution of acute inflammatory changes, contrasting with persistent chronic skin abnormalities.

Dermatologic examination revealed diffuse cutaneous involvement of the bilateral lower extremities characterized by irregularly distributed hyperpigmented and hypopigmented patches with areas of induration (Figure 1). In addition, there was superimposed erythema and warmth of the right lower extremity with poorly demarcated borders, concerning for a concurrent inflammatory or infectious process (Figure 2). A skin biopsy was ordered to confirm the diagnosis.

Diagnosis and management

Dermatologic evaluation raised a high clinical suspicion for cutaneous T-cell lymphoma, specifically MF, based on the presence of chronic, diffuse, pruritic patches and plaques involving the trunk and extremities. However, multiple skin biopsies obtained from different anatomic sites consistently demonstrated subacute spongiotic dermatitis with focal lymphocytic exocytosis. There was no definitive epidermotropism, cytologic atypia, or presence of cerebriform lymphocytes to support a diagnosis of cutaneous T-cell lymphoma. Immunohistochemical analysis revealed a predominantly CD3-positive T-cell infiltrate with a preserved CD4:CD8 ratio and retained CD7 expression, without evidence of an aberrant T-cell phenotype. Overall, these findings were nonspecific and overlapped with inflammatory dermatoses, limiting diagnostic confirmation.

Direct immunofluorescence studies were negative, excluding an immunobullous process. Peripheral blood flow cytometry likewise did not identify an abnormal T-cell population or evidence of a clonal lymphoproliferative disorder. T-cell receptor (TCR) gene rearrangement studies were not performed during this evaluation. Given the absence of definitive histopathologic or immunophenotypic abnormalities, along with the patient’s clinical stability, further molecular testing was deferred in favor of close clinical monitoring and consideration of repeat biopsy if lesions evolved.

Cross-sectional imaging of the chest, abdomen, and pelvis did not demonstrate definitive systemic or visceral involvement. A right external iliac lymph node was identified but considered nonspecific, with differential considerations including reactive versus early involvement, as there was no additional lymphadenopathy or radiographic evidence of advanced disease.

Given the discordance between the patient’s clinical presentation and histopathologic findings, the working diagnosis remained suspected early-stage cutaneous T-cell lymphoma (MF) versus chronic inflammatory dermatitis. Multidisciplinary discussion emphasized the importance of continued clinical monitoring and the potential need for repeat biopsy to improve diagnostic yield if lesions evolved.

Concurrently, the patient’s acute right lower extremity erythema, warmth, and swelling were managed as cellulitis. She was initially treated with intravenous vancomycin and cefepime, with subsequent de-escalation to vancomycin monotherapy during hospitalization. A nasal swab for methicillin-resistant *Staphylococcus aureus* returned positive. The patient demonstrated clinical improvement, and she was transitioned to oral doxycycline (100 mg twice daily) to complete a 14-day course of therapy.

Management of the chronic cutaneous process focused on high-potency topical corticosteroids, with plans for close outpatient dermatologic follow-up and consideration of systemic therapy, including methotrexate, pending clinical course and further diagnostic clarification.

Multidisciplinary board meeting

Given the diagnostic uncertainty, the case was reviewed in a multidisciplinary setting including dermatology, hematology/oncology, radiology, and internal medicine. Discussion focused on the clinical suspicion for cutaneous T-cell lymphoma, staging considerations, and the significance of a right external iliac lymph node.

Imaging did not demonstrate definitive systemic or visceral involvement, and there was no evidence of advanced nodal disease. The identified lymph node was considered nonspecific, with differential including reactive versus early involvement.

The group emphasized the importance of clinicopathologic correlation in the setting of persistent clinical suspicion despite nondiagnostic biopsies. At the time of evaluation, findings favored predominantly cutaneous disease versus chronic inflammatory dermatitis without clear systemic involvement.

Recommendations included close clinical follow-up, repeat biopsy of evolving lesions to improve diagnostic yield, and consideration of further staging if clinically indicated. Management focused on dermatologic-directed therapy with coordination of hematology/oncology if diagnostic confirmation was achieved. The patient’s delayed presentation and lack of prior definitive diagnosis were also noted, underscoring the potential impact of social determinants of health on timely evaluation.

Clinical outcome

During hospitalization, the patient demonstrated clinical stabilization with improvement in pain and soft tissue symptoms following initiation of treatment. No acute complications were identified, and there was no radiographic evidence of rapidly progressive systemic disease.

The patient was discharged with close follow-up arranged with dermatology and hematology/oncology for ongoing management of suspected MF. This case underscores a delayed diagnosis in the setting of limited healthcare access, with disease recognition occurring only after hospital admission, despite likely longstanding cutaneous involvement.

## Discussion

Background (history, epidemiology, risk factors)

MF is the most common subtype of cutaneous T-cell lymphoma, characterized by a malignancy of skin-homing CD4+ T lymphocytes. Classified under primary cutaneous lymphomas according to the World Health Organization-European Organization for Research and Treatment of Cancer (WHO-EORTC) system, MF typically follows an indolent course, progressing from patches to plaques and eventually tumors [4,5]. Despite this generally slow progression, early-stage MF often resembles benign inflammatory dermatoses such as eczema or psoriasis, which can result in significant delays in diagnosis [3,6].

The pathogenesis of MF involves chronic antigenic stimulation and immune dysregulation, which may compromise the skin barrier and increase susceptibility to secondary complications. In advanced or untreated cases, this impaired barrier function may predispose patients to superimposed bacterial infections, such as cellulitis, which can obscure the underlying malignancy and further complicate the clinical picture [3,4]. As a result, the diagnosis of MF may be delayed until secondary complications, rather than the primary disease itself, prompt medical evaluation. Additionally, socioeconomic barriers and limited access to care may further contribute to delayed recognition, as patients often present only when symptoms become severe.

Here, we present a case of suspected MF in a 64-year-old female whose initial presentation was dominated by progressive right lower extremity pain and swelling concerning cellulitis. This case illustrates the diagnostic “eclipse” created by secondary soft tissue infection and highlights how social determinants of health can contribute to delayed recognition of cutaneous malignancies. This case highlights the importance of maintaining a high index of suspicion for cutaneous T-cell lymphoma in patients presenting with atypical or treatment-refractory skin findings, even when masked by acute infectious processes. Distinguishing clinical features between cellulitis and MF, synthesized from prior literature describing the clinical presentation, diagnostic challenges, and mimicking nature of MF, are summarized in Table 2 [3,7,8].

**Table 2:** Key clinical and diagnostic features distinguishing cellulitis from mycosis fungoides, emphasizing differences in onset, progression, morphology, and response to therapy.

Feature	Cellulitis	Mycosis fungoides
Onset	Acute	Chronic, indolent
Clinical course	Rapid progression over days	Slowly progressive over months to years
Skin findings	Erythema, warmth, tenderness	Patches, plaques, pigmentary changes
Mimicry potential	—	Frequently mimics benign dermatoses
Systemic features	May include fever, leukocytosis	Typically absent in early disease
Laboratory findings	Elevated inflammatory markers	Often nonspecific or normal
Response to therapy	Improves with antibiotics	Often persistent or treatment-refractory
Key diagnostic approach	Clinical diagnosis	Requires clinicopathologic correlation
Definitive diagnosis	Not required	Skin biopsy
Histopathology	Not applicable	Epidermotropism, atypical lymphocytes with cerebriform nuclei

Physiology and pathophysiology

MF is the most common cutaneous T-cell lymphoma and classically presents as T cells with cerebriform nuclei. MF is thought to arise in the setting of malignant transformation of skin-homing CD4+ T lymphocytes. Although the exact pathogenesis remains incompletely understood, chronic antigenic stimulation has been proposed as a contributing mechanism, leading to persistent activation and proliferation of T cells, which may promote the accumulation of genetic abnormalities and genomic instability [12]. These alterations include deletions of tumor suppressor genes such as *CDKN2A* and dysregulation of signaling pathways, including NF-κB and JAK/STAT [12]. In more advanced disease, *TP53* mutations can also occur, further contributing to clonal expansion and disease progression [12].

Building on these genetic and cellular changes, malignant CD4+ T cells in MF exhibit epidermotropism, or abnormal migration to the epidermis. There, they may form Pautrier microabscesses that disrupt skin architecture and cause inflammation, resulting in flat, scaly, often pruritic patches of varying sizes [4,13]. These patches may thicken into plaques and develop into tumors over time [4,13]. As MF progresses, CD4+ cell accumulation can contribute to lymphatic obstruction and immune dysregulation, which may manifest as lymph node enlargement [13].

As MF advances, a shift toward a Th2 cytokine profile contributes to impaired cell-mediated immunity [4]. This immune dysregulation, in combination with a compromised skin barrier, increases susceptibility to secondary bacterial infections [4,8]. Disruption of the epidermal barrier facilitates microbial entry, while impaired immune surveillance reduces the ability to control bacterial proliferation. Together, these mechanisms increase vulnerability to infections such as cellulitis, most commonly caused by *Staphylococcus aureus* and β-hemolytic *Streptococcus pyogenes* [8].

Comparative analysis of this case with current literature (clinical presentation, diagnostic workup, management, outcome)

MF typically presents as an indolent cutaneous lymphoma characterized by slowly progressive erythematous patches and plaques, most frequently affecting non-sun-exposed areas such as the trunk, buttocks, and upper thighs [14]. These lesions are often accompanied by pruritus, the most common presenting symptom, reported in up to 61% of patients with cutaneous lymphoma [15]. Because these findings closely resemble benign inflammatory dermatoses, diagnosis is frequently delayed and often requires repeated clinical evaluations and serial biopsies over time [16]. This delay is well documented, with prospective registry data from the PROCLIPI study reporting a median time to diagnosis of approximately 36 months [17]. In the same cohort, over half of patients were diagnosed in the outpatient setting, reflecting the typically gradual and nonacute nature of disease recognition. Similar findings have been reported in other cohorts, reinforcing that delayed diagnosis is a common and clinically significant feature of MF [18]. In contrast to this gradual and insidious course, our patient presented acutely with painful soft tissue inflammation of the lower extremity, initially concerning for cellulitis, representing a misleading clinical manifestation. This atypical presentation led to inpatient evaluation, during which suspicion of an underlying skin condition was first raised despite no prior recognition of the disease. The absence of earlier evaluation suggests limited engagement with the healthcare system, which may have contributed to delayed recognition of the underlying condition. Barriers to care and other social determinants of health can further limit access to timely dermatologic evaluation, particularly when atypical inflammatory features obscure the clinical picture.

The diagnostic evaluation of suspected MF is often limited by nonspecific findings, particularly in early-stage disease, where imaging primarily aids staging rather than diagnosis [5]. In this case, cross-sectional imaging demonstrated no visceral involvement, and a solitary external iliac lymph node without additional lymphadenopathy was interpreted cautiously. This approach is consistent with literature indicating that early disease is typically confined to the skin and that isolated nodal abnormalities require clinical and pathologic correlation before being considered evidence of disease progression [4,5].

Histopathologic confirmation remains the gold standard; however, early MF frequently lacks definitive diagnostic features and may instead demonstrate nonspecific patterns such as spongiotic dermatitis or superficial lymphocytic infiltrates, as observed in this patient [3,4]. Classic findings such as epidermotropism and atypical lymphocytes with cerebriform nuclei may be absent or subtle in early disease, contributing to inconclusive biopsy results [3,14]. Ancillary diagnostic techniques include immunohistochemical analysis of T-cell markers and assessment of T-cell receptor gene rearrangements. In classic presentations, MF often demonstrates loss of pan-T-cell markers such as CD7 and an elevated CD4:CD8 ratio. Detection of T-cell monoclonality through receptor gene rearrangement studies can further support the diagnosis of T-cell lymphoma. However, these features are not consistently present in early-stage disease and may be absent, as demonstrated in this case [4,14]. When immunophenotypic abnormalities are lacking, and T-cell receptor studies demonstrate a polyclonal pattern, diagnostic certainty is reduced. In this setting, the differential diagnosis remains broad and includes benign inflammatory dermatoses such as eczema and psoriasis, as well as infectious processes such as cellulitis [3]. In our patient, the acute presentation with erythema, warmth, and swelling appropriately prompted treatment for cellulitis, reflecting a diagnostic challenge in which a superimposed infection obscured recognition of a possible underlying cutaneous T-cell lymphoma [3,8]. This discrepancy between clinical findings and histopathology necessitates integration of clinical, pathologic, and longitudinal data rather than reliance on a single diagnostic evaluation [4,14].

Management of suspected MF is guided by disease stage and diagnostic certainty, with early-stage or indeterminate cases typically managed conservatively using skin-directed therapies and close clinical surveillance [4,5]. First-line treatment in confirmed early-stage disease includes topical corticosteroids, phototherapy, or other skin-directed modalities, reflecting the indolent course and emphasis on symptom control [4,14]. In this case, the absence of definitive histopathologic confirmation appropriately limited management to topical corticosteroids with close outpatient follow-up, consistent with recommendations supporting ongoing reassessment in diagnostically uncertain presentations [3,14].

Concurrently, the patient’s acute lower extremity erythema and swelling were treated with intravenous antibiotics for cellulitis, resulting in clinical improvement. This approach aligns with literature describing increased susceptibility to bacterial infections in MF due to impaired skin barrier function and immune dysregulation [4,8], while inflammatory or infectious processes may complicate recognition of underlying disease [3]. Avoiding premature escalation to systemic therapy is consistent with guideline-based management, which reserves such interventions for advanced or refractory disease and supports observation in early or uncertain cases [5,19].

Outcomes in MF vary by stage, with early disease generally associated with a favorable prognosis and prolonged survival, whereas advanced stages are associated with an increased risk of progression and systemic involvement [4,5]. In contrast to the typical longitudinal course, the short-term outcome in this patient was limited to clinical stabilization, with improvement in acute inflammatory symptoms but no resolution of the underlying cutaneous process. When histopathologic findings remain nondiagnostic, prognosis cannot be reliably determined at a single time point, and early disease may not show clear progression or confirmation during a single encounter [3,14].

What we learned from this case

This case illustrates a critical diagnostic tension in dermatology and internal medicine: the coexistence of an acute, treatable condition can obscure recognition of an underlying, indolent malignancy. While the patient’s presenting symptoms were consistent with cellulitis and appropriately improved with antibiotic therapy, the persistence of chronic, diffuse cutaneous abnormalities reflected a separate and unresolved disease process.

A key learning point is that clinical improvement of an acute process should not prematurely close the diagnostic evaluation, particularly when additional findings fall outside the expected disease trajectory. In this case, cellulitis represented a superimposed condition rather than the primary diagnosis, and anchoring on infection risked overlooking a possible cutaneous T-cell lymphoma.

Importantly, this case emphasizes the concept of clinicopathologic discordance. Despite strong clinical suspicion for MF, repeated biopsies and immunophenotypic studies remained nondiagnostic. Early-stage disease may lack definitive histologic features, requiring longitudinal reassessment rather than reliance on a single biopsy result.

This case also reinforces that MF is ultimately a clinical-pathologic diagnosis made over time, not a single test-based diagnosis. Persistent or evolving lesions, especially those that are treatment-refractory or morphologically atypical, warrant continued surveillance and repeat tissue sampling.

Finally, the patient’s delayed presentation highlights the role of healthcare access in oncologic outcomes. Limited engagement with the healthcare system contributed to prolonged diagnostic uncertainty, underscoring the importance of early dermatologic evaluation in patients with chronic unexplained skin disease.

## Conclusions

This case highlights the diagnostic challenge of distinguishing cellulitis from its mimickers, particularly when an acute inflammatory process coexists with a chronic and potentially malignant dermatologic condition. While the patient’s acute symptoms responded to antibiotic therapy, the persistence of longstanding cutaneous abnormalities raised concern for an underlying process such as MF, despite nondiagnostic histopathologic and immunophenotypic findings. This case underscores the importance of maintaining diagnostic vigilance in patients with atypical or chronic skin findings that do not fully align with an infectious process. In cases of persistent clinicopathologic discordance, clinicians should consider repeat biopsy of evolving or untreated lesions, particularly when clinical suspicion remains high despite initial nondiagnostic studies. Early-stage MF may evade definitive diagnosis on initial evaluation, necessitating longitudinal assessment and integration of clinical, histologic, and ancillary data over time. Recognizing patterns of clinicopathologic discordance and avoiding premature diagnostic closure are essential to preventing delays in the identification of cutaneous T-cell lymphoma. Increased awareness of this presentation may facilitate earlier recognition, appropriate specialist referral, and improved patient outcomes.
